# Clinical Efficacy and Safety of Mesenchymal Stem Cells for Systemic Lupus Erythematosus

**DOI:** 10.1155/2020/6518508

**Published:** 2020-04-03

**Authors:** Tianbiao Zhou, Hong-Yan Li, Chunling Liao, Wenshan Lin, Shujun Lin

**Affiliations:** ^1^Department of Nephrology, The Second Affiliated Hospital, Shantou University Medical College, 515041 Shantou, China; ^2^Department of Nephrology, Huadu District People's Hospital of Guangzhou, Southern Medical University, 510800 Guangzhou, China

## Abstract

Systemic lupus erythematosus (SLE) is a polymorphic, multisystemic autoimmune disease that causes multiorgan damage in which cellular communication occurs through the involvement of autoantibodies directed against autoantigen production. Mesenchymal stem cells (MSCs), which have strong protective and immunomodulatory abilities, are obtained not only from bone marrow but also from medical waste such as adipose tissue and umbilical cord tissue and have been recognized as a promising tool for the treatment of various autoimmune diseases and inflammatory disorders. This meta-analysis is aimed at assessing whether MSCs can become a new treatment for SLE with good efficacy and safety. Based on predetermined criteria, a bibliographical search was performed from January 1, 2000, to July 31, 2019, by searching the following databases: ISI Web of Science, Embase, PubMed, the Cochrane Library, and the Chinese Biomedical Literature Database (CBM). Eligible studies and data were identified. Statistical analysis was conducted to assess the efficacy (proteinuria, systemic lupus erythematosus disease activity index (SLEDAI), Scr, BUN, albumin, C3, and C4) and safety (rate of adverse events) of MSCs for SLE using Cochrane Review Manager Version 5.3. Ten studies fulfilled the inclusion criteria and were eligible for this meta-analysis, which comprised 8 prospective or retrospective case series and four randomized controlled trails (RCTs) studies. In the RCT, the results indicated that the MSC group had lower proteinuria than the control group at 3 months and 6 months and the MSC group displayed a lower SLEDAI than the control group at 2 months and 6 months. Furthermore, the MSC group showed a lower rate of adverse events than the control group (OR = 0.26, 95% CI: 0.07, 0.89, *P* = 0.03). In the case series trials, the results indicated that the MSC group had lower proteinuria at 1 month, 2 months, 3 months, 4 months, 6 months, and 12 months. In conclusion, MSCs might be a promising therapeutic agent for patients with SLE.

## 1. Introduction

Mesenchymal stem cells (MSCs) are a group of self-renewing nonhematopoietic multipotent progenitor cells that were initially discovered in bone marrow and subsequently found in many other tissues, such as umbilical cord blood, adipose tissue, skin tissue, and the periendothelial area. They can differentiate into various types of mesenchymal cells, such as osteoblasts, chondrocytes, fibroblasts, and adipocytes [1, 2]. To date, the cells have been mainly defined retrospectively based on their fibroblastic colony-forming capacity and multipotency in vitro. Therefore, these cells have been redefined as MSCs. It has been shown that MSCs have unique and powerful immunomodulatory and regenerative characteristics. The therapeutic effects of MSCs can be largely attributed to extracellular vesicles including exosomes. Exosomes from MSCs can regulate the inflammatory response, immunomodulation, angiogenesis, blood coagulation, extracellular matrix remodelling, and cell apoptosis; moreover, exosomes can also reduce the levels of creatinine (Cr) and blood urea nitrogen (BUN), as well as necrosis of proximal kidney tubules [3–5]. MSC transplantation has become one of the treatment options for a variety of immune system diseases, such as multiple sclerosis (MS) and systemic lupus erythematosus (SLE) [1, 6–8].

As a chronic autoimmune disease, systemic lupus erythematosus (SLE) is accompanied by multiple system damage. Immune-mediated inflammatory injury plays an important role in the pathogenesis of SLE. The disease is characterized by the production of a variety of autoantibodies represented by antinuclear antibodies, the formation of immune complexes, tissue inflammation in multiple organs (including brain, joints, blood vessels, kidneys, and skin), and high levels of serum proinflammatory cytokines [9, 10]. Lupus nephritis (LN) is one of the most serious visceral complications in SLE, occurring in approximately half of SLE patients. Clinically, LN is characterized by proteinuria, cellular casts, haematuria, and renal failure, which may lead to end-stage renal disease and the need for peritoneal dialysis, haemodialysis, or renal transplantation [11]. At present, the main drugs for treating SLE include antimalarial drugs (hydroxychloroquine (HCQ), quinacrine), corticosteroids and nonsteroidal anti-inflammatory drugs (NSAIDs), immunosuppressants (cyclosporine A (CsA), tacrolimus (TAC), methotrexate (MTX), azathioprine (AZA), mycophenolate mofetil (MMF), and cyclophosphamide (CTX)), and biological agents (belimumab antibody, rituximab (RTX)) [12]. When the clinical condition is serious, high-dose immunoglobulin, plasma exchange, or haematopoietic stem cell or mesenchymal stem cell transplantation can be selected. However, the long-term use of corticosteroids or immunosuppressants may lead to serious infection and secondary malignant tumours, and the use of biological agents is also limited to a certain extent because of its high cost [13].

Previously, there were some studies focusing on the MSC in treating renal diseases, and the results were conflicting. Quimby et al. [14] conducted a study in cats with chronic kidney disease and reported that administration of MSCs was not associated with significant improvement in renal function. van Rhijn-Brouwer et al. [15] conducted a study in kidney transplant recipients and showed that MSCs have an intrinsic capacity to produce proangiogenic paracrine factors, including extracellular vesicles (EVs), which suggested that autologous MSC-based therapy is a viable option in the therapy of chronic kidney disease. Song et al. [16] reported that MSC treatment can attenuate renal interstitial fibrosis possibly through inhibition of EMT and the inflammatory response via the TGF-*β*1 signalling pathway.

Cell therapy has become an attractive therapeutic strategy for various types of diseases [17–20], and it has achieved certain curative effects in induction therapy in patients with SLE [13]. The purpose of this study was to evaluate the efficacy of MSCs in the treatment of SLE by meta-analysis.

## 2. Materials and Methods

### 2.1. Data Sources and Search Terms

The previous full extent of studies from January 1, 2000, to July 31, 2019, reporting the outcomes of MSC treatment for LN patients had been mined in this search strategy to determine the therapeutic promise of MSC regimen for LN as it was translated from bench to bedside. Two reviewers separately conducted the searches in the following medical databases: ISI Web of Science, Embase, PubMed, the Cochrane Library, and the Chinese Biomedical Literature Database (CBM). PubMed was searched using MeSH headings or their equivalents of “Mesenchymal Stem Cells” and “Lupus Nephritis.” The entry terms for mesenchymal stem cells were as follows: Mesenchymal Stem Cells, MSC, Multipotent Stromal Cells, Mesenchymal Stromal Cells, Mesenchymal Progenitor Cells, Wharton Jelly Cells, Adipose-Derived Mesenchymal Stem Cells, and Bone Marrow Stromal Stem Cells. The entry terms for SLE were as follows: systemic lupus erythematosus, SLE, Lupus Nephritis, LN, Lupus Glomerulonephritis, Lupus Nephritides, and Lupus Glomerulonephritides. As per this method, other database searches were performed using a combination of mesenchymal stem cells and lupus nephritis terms. Any language restrictions were not applied in this meta-analysis. Additionally, the reference lists of the selected studies and reviews were also scrutinized to manually identify eligible articles.

### 2.2. Inclusion and Exclusion Criteria

The inclusion criteria are as follows: (1) eligible articles were required to be randomized controlled trials (RCTs) or self-controlled trials; (2) enrolled patients were diagnosed with LN disease conforming to the American College of Rheumatology (ACR) criteria and were treated with MSC therapy; (3) the presence of data on therapeutic efficacy and safety was essential.

The exclusion criteria are as follows: (1) abstracts, case reports, reviews, case-controlled trials, and editorials were excluded; (2) patient data that were not shown or were not sufficiently detailed to be pooled were excluded.

### 2.3. Study Selection and Data Extraction

Titles, abstracts, and, if necessary, full texts were browsed by two independent investigators. Discrepancies were resolved by them through comparing lists after reviewing the identified papers, and another investigator finalized the list of included articles.

Two investigators customized a table to extract the data independently on the basis of the surname of the first author, the publication year, patient information, the intervention, and the outcome characteristics. Any disagreement was settled by a third investigator.

### 2.4. Statistical Analysis

All statistical analyses were carried out by Cochrane Review Manager Version 5.3 (Cochrane Library, UK). *I*^2^ was used to detect the heterogeneity among the included investigations. A random effects model was applied for meta-analyses, in which the *P* value from the heterogeneity test was less than 0.1; otherwise, a fixed effects model was used. Weighted mean differences (WMDs) were presented for continuous data, and the binary data were shown for odds ratios (ORs). 95% confidence intervals (95% CIs) were assessed for the recruited studies. Values of *P* < 0.05 were considered statistically significant.

## 3. Results

### 3.1. Search Results

**Figure 1:**
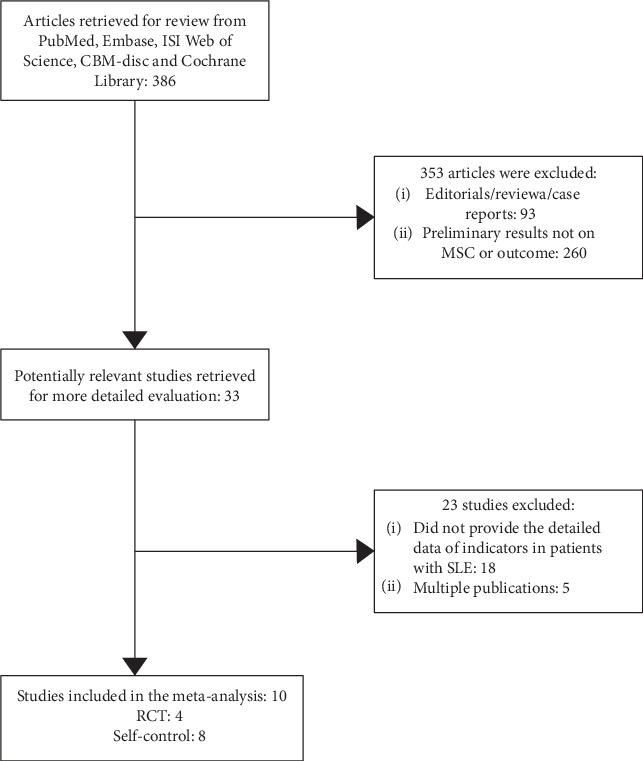
Flow diagram process of study selection.

The searches identified 386 publications, and 10 studies fulfilled the inclusion criteria and were eligible for this meta-analysis, which comprised 8 prospective or retrospective case series [21–28] and four RCT studies [27–30] (Figure 1). These eight retrospective or prospective case series included 231 SLE patients, as shown and detailed in Table 1. Furthermore, these four RCTs included 47 patients with SLE in the case group and 37 patients with SLE in the control group.

### 3.2. Randomized Controlled Trial

#### 3.2.1. Proteinuria

One study [28] was included in the meta-analysis for 3 months and two [28, 30] for 6 months, and the results indicated that the MSC group had lower proteinuria than the control group (3 months: WMD = ‐0.92, 95% CI: -1.05, -0.79, *P* < 0.00001; 6 months: WMD = ‐2.00, 95% CI: -3.81, -0.19, *P* = 0.03; Table 2). However, one study [28] was included for 2 months and two studies [27, 29] were included for 12 months. The results indicated that MSC treatment resulted in lower proteinuria, but the difference was not significant (2 months: WMD = ‐1.74, 95% CI: -5.00, -1.52, *P* = 0.30; 12 months: WMD = ‐0.46, 95% CI: -1.37, 0.45, *P* = 0.33; Table 2).

#### 3.2.2. Scr

One study [27] was included for 3 months, one [30] for 6 months, and two [27, 29] for 12 months, and the results indicated that the difference between the MSC treatment group and the control group was not notable (3 months: WMD = ‐2.52, 95% CI: -8.53, 3.49, *P* = 0.41; 6 months: WMD = 3.92, 95% CI: -8.55, 16.39, *P* = 0.54; and 12 months: WMD = ‐0.74, 95% CI: -14.04, 12.56, *P* = 0.91; Table 2).

#### 3.2.3. Serum Albumin

One study [27] was included in the meta-analysis for 3 months, and the results indicated that the MSC group had higher serum albumin than the control group (WMD = 7.85, 95% CI: 5.93, 9.77, *P* < 0.00001; Table 2). However, two studies [27, 29] were included for 12 months, and the results indicated that MSC treatment resulted in higher serum albumin, but the difference was not significant (WMD = 0.94, 95% CI: -0.53, 2.40, P = 0.21; Table 2).

#### 3.2.4. C3

One study [27] was included in the meta-analysis for 3 months, and the results indicated that the MSC group had higher C3 than the control group (WMD = 0.28, 95% CI: 0.16, 0.40, *P* < 0.00001; Table 2). However, two studies [27, 29] were included for 12 months and the results indicated that MSC treatment resulted in higher C3, but the difference was not significant (WMD = 0.36, 95% CI: -0.08, 0.79, *P* = 0.11; Table 2).

#### 3.2.5. C4

One study [27] was included for 3 months and two [27, 29] for 12 months, and the results indicated that the difference was not significant between the MSC treatment group and the control group (3 months: WMD = ‐0.01, 95% CI: -0.04, 0.02, *P* = 0.46; 12 months: WMD = ‐0.01, 95% CI: -0.03, 0.01, *P* = 0.39; Table 2).

#### 3.2.6. SLEDAI

One study [28] was included in the meta-analysis for 2 months and one [28] for 6 months, and the results indicated that the MSC group had a lower SLEDAI than the control group (2 months: WMD = ‐6.25, 95% CI: -9.04, -3.46, *P* < 0.0001; 6 months: WMD = ‐4.25, 95% CI: -6.78, -1.72, *P* = 0.001; Table 2). However, one study [27] was included for 3 months and two studies [27, 29] were included for 12 months. The results indicated that MSC treatment resulted in a lower SLEDAI, but the difference was not significant (3 months: WMD = ‐0.89, 95% CI: -2.19, 0.41, *P* = 0.18; 12 months: WMD = ‐1.00, 95% CI: -3.13, 1.14, *P* = 0.36; Table 2).

#### 3.2.7. Adverse Events

Two studies [28, 30] were included in the meta-analysis for adverse events. The adverse events included upper respiratory tract infection, leucopenia, pneumonia, and subcutaneous abscess in the MSC group and included upper respiratory tract infection, stroke, and ascites in the control group. The results indicated that the MSC group had a lower rate of adverse events than the control group (OR = 0.26, 95% CI: 0.07, 0.89, *P* = 0.03; Table 2).

### 3.3. Case Series

#### 3.3.1. Proteinuria

**Figure 2:**
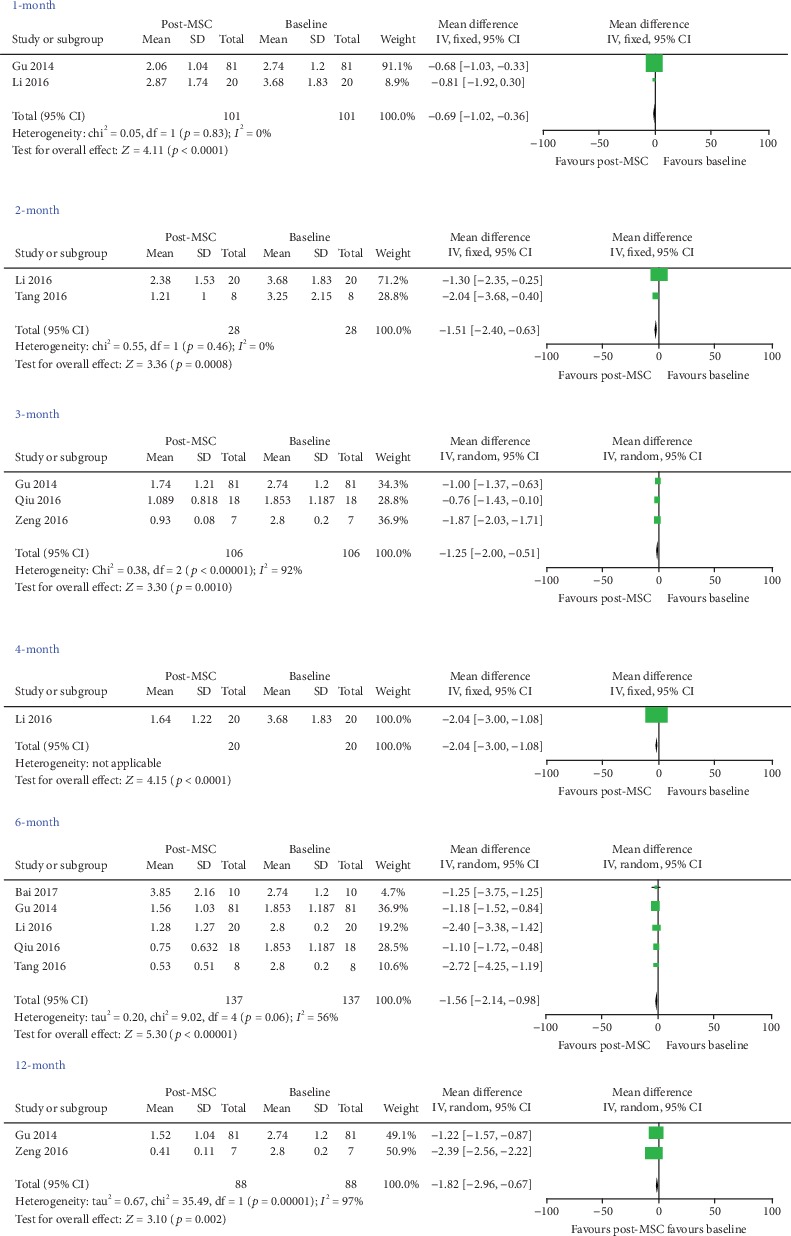
Assessment the efficacy of MSC on proteinuria in patients with systemic lupus erythematosus (self-controlled studies).

Two studies [21, 22] were included for the meta-analysis for 1 month, and the results indicated that the MSC group had lower proteinuria (WMD = ‐0.69, 95% CI: -1.02, -0.36, *P* < 0.0001; Figure 2 and Table 3). Two studies [22, 28] were included in the meta-analysis for 2 months, and the results indicated that the MSC group had better efficacy (WMD = ‐1.51, 95% CI: -2.40, -0.63, *P* = 0.0008; Figure 2 and Table 3). Three studies [21, 25, 27] were included in the meta-analysis for 3 months, and the results indicated that the MSC group had lower proteinuria (WMD = ‐1.25, 95% CI: -2.00, -0.51, *P* = 0.001; Figure 2 and Table 3). One study [22] was included in the meta-analysis for 4 months, and the results indicated that the MSC group had better efficacy (WMD = ‐2.04, 95% CI: -3.00, -1.08, *P* < 0.0001; Figure 2 and Table 3). Five studies [21, 22, 24, 25, 28] were included in the meta-analysis for 6 months, and the results indicated that the MSC group had lower proteinuria (WMD = −1.56, 95% CI: -2.14, -0.98, *P* < 0.00001; Figure 2 and Table 3). Two studies [21, 27] were included in the meta-analysis for 12 months, and the results indicated that the MSC group had reduced proteinuria (WMD = ‐1.82, 95% CI: -2.96, -0.67, *P* = 0.002; Figure 2 and Table 3).

#### 3.3.2. Scr

Three studies [21–23] were included for 1 month, two [22, 23] for 2 months, three [21, 23, 27] for 3 months, and one [22] for 4 months, and the results indicated that MSC treatment yielded a better reduction in Scr but the difference was not significant (1 month: WMD = ‐7.28, 95% CI: -21.97, 7.41, *P* = 0.33; 2 months: WMD = ‐59.18, 95% CI: -166.92, 48.56, *P* = 0.28; 3 months: WMD = −75.13, 95% CI: -187.01, 36.76, *P* = 0.19; and 4 months: WMD = ‐10.25, 95% CI: -25.34, 4.84, *P* = 0.18; Table 3). Interestingly, two studies [21, 22] were included for 6 months and two studies [21, 27] for 12 months, and the results indicated that MSC treatment resulted in lower Scr (6 months: WMD = ‐14.08, 95% CI: -28.09, -0.07, *P* = 0.05; 12 months: WMD = ‐30.00, 95% CI: -38.89, -21.10, *P* < 0.00001; Table 3).

#### 3.3.3. BUN

One study [23] was included for 1 month, one [23] for 2 months, and two [21, 27] for 12 months, and the results indicated that MSC treatment yielded a lower BUN (1 month: WMD = ‐610.60, 95% CI: -835.84, -385.36, *P* < 0.00001; 2 months: WMD = ‐758.40, 95% CI: -960.42, -556.38, *P* < 0.00001; and 12 months: WMD = ‐4.14, 95% CI: -7.89, -0.39, *P* = 0.03; Table 3). However, three studies [21, 23, 27] were included for 3 months, and the results indicated that MSC treatment had better efficacy, but the difference was not significant (WMD = ‐21.31, 95% CI: -46.58, 3.97, *P* = 0.10; Table 3).

#### 3.3.4. C3

Two studies [22, 23] were included for 1 month, two [22, 23] for 2 months, one [22] for 4 months, three [22, 24, 25] for 6 months, and one [27] for 12 months, and the results indicated that MSC treatment resulted in a higher level of C3 (1 month: WMD = 0.15, 95% CI: 0.06, 0.24, *P* = 0.0006; 2 months: WMD = 0.25, 95% CI: 0.17, 0.33, *P* < 0.00001; 4 months: WMD = 0.33, 95% CI: 0.13, 0.53, *P* = 0.001; 6 months: WMD = 0.23, 95% CI: 0.06, 0.39, *P* = 0.006; and 12 months: WMD = 0.96, 95% CI: 0.88, 1.04, *P* < 0.00001; Table 3). However, three studies [23, 25, 27] were included for 3 months, and the results indicated that MSC treatment increased the C3 levels but the difference was not significant (WMD = 0.37, 95% CI: -0.01, 0.76, *P* = 0.06; Table 3).

#### 3.3.5. C4

Two studies [22, 23] were included for 2 months, two [23, 27] for 3 months, one [22] for 4 months, and one [27] for 12 months, and the results indicated that MSC treatment resulted in a higher level of C4 (2 months: WMD = 0.05, 95% CI: 0.02, 0.08, *P* = 0.0001; 3 months: WMD = 0.11, 95% CI: 0.07, 0.15, *P* < 0.00001; 4 months: WMD = 0.07, 95% CI: 0.04, 0.10, *P* < 0.0001; and 12 months: WMD = 0.24, 95% CI: 0.22, 0.26, *P* < 0.00001; Table 3). However, two studies [22, 23] were included for 1 month, and two studies [22, 24] were included for 6 months. The results indicated that MSC treatment increased the C4 level, but the difference was not significant (1 month: WMD = 0.02, 95% CI: -0.01, 0.04, *P* = 0.25; 6 months: WMD = 0.06, 95% CI: -0.02, 0.14, *P* = 0.15; Table 3).

#### 3.3.6. SLEDAI

**Figure 3:**
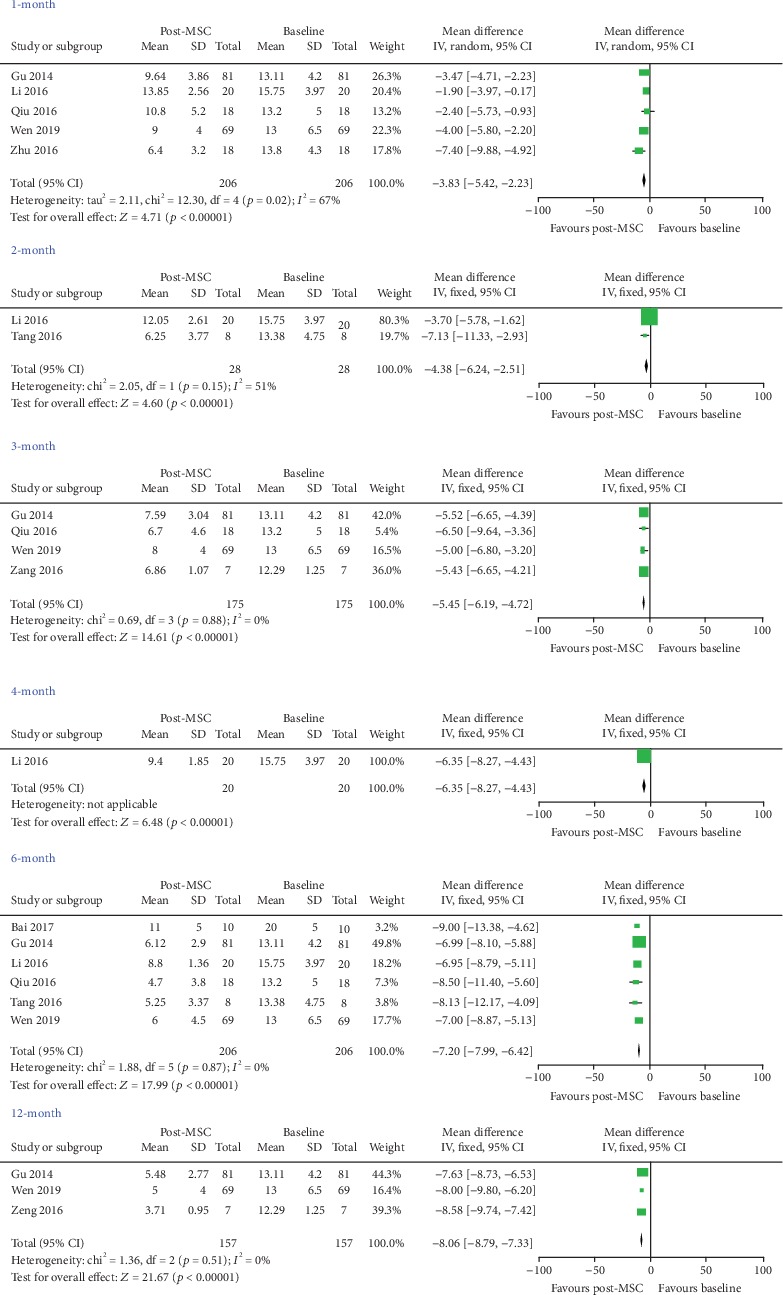
Assessment the efficacy of MSC on SLEDAI in patients with systemic lupus erythematosus (self-controlled studies).

Five studies [21–23, 25, 26] were included for 1 month, two [22, 28] for 2 months, four [21, 25–27] for 3 months, one [22] for 4 months, six [21, 22, 24–26, 28] for 6 months, and three [21, 26, 27] for 12 months, and the results indicated that MSC treatment yielded a lower value of SLEDAI (1 month: WMD = ‐3.83, 95% CI: -5.42, -2.23, *P* < 0.00001; 2 months: WMD = ‐4.38, 95% CI: -6.24, -2.51, *P* < 0.00001; 3 months: WMD = ‐5.45, 95% CI: -6.19, -4.72, *P* < 0.00001; 4 months: WMD = ‐6.35, 95% CI: -8.27, -4.43, *P* < 0.00001; 6 months: WMD = ‐7.20, 95% CI: -7.99, -6.42, *P* < 0.00001; and 12 months: WMD = ‐8.06, 95% CI: -8.79, -7.33, *P* < 0.00001; Figure 3 and Table 3).

## 4. Discussion

In this study, the meta-analysis included two parts, one for RCT studies and one for self-controlled studies. All the included studies found that MSC treatment can achieve better efficacy, except for the investigation from Tang et al. [28]. In this meta-analysis of RCTs, the results indicated that MSC treatment can achieve better efficacy than the control treatment at 3 months, with results such as lower proteinuria, increased serum albumin, and increased serum C3. MSC treatment resulted in lower SLEDAI values at 3 months and 6 months. Furthermore, the rate of adverse events in the MSC group was lower than that in the control group. The data from the meta-analysis of RCTs indicated that MSC treatment might be a good treatment for SLE, but the sample size of the recruited investigations was small, and the results should thus be carefully examined.

Furthermore, a meta-analysis including self-controlled studies was also conducted, and the results indicated that MSC treatment can markedly reduce proteinuria and the value of SLEDAI at 1 month, 2 months, 3 months, 4 months, 6 months, and 12 months. It can also improve the values of Scr, BUN, C3, and C4 at some time points. MSCs might be a good treatment agent for SLE in the clinic. More studies with larger sample sizes should be conducted to confirm these findings in the future.

In the included studies, Zeng et al. [27] conducted an RCT and recruited 22 patients with LN, and the results indicated that MSC combined with MMF in the treatment of LN can quickly reduce urinary protein in the short term and play a protective role in renal function, which can remarkably improve the disease condition and reduce the recurrence rate. Yang et al. [29] also found that it was effective and safe for SLE refractory to UC-MSC treatment. However, the RCT from Tang et al. [28] reported that the clinical symptoms and laboratory examination results of patients in two teams were all improved, but there was no notably significant difference between the two teams. Deng et al. [30] also indicated that MSC for SLE patients has no apparent additional effect over and above standard immunosuppression from their RCT study. Interestingly, all the self-controlled studies [21–26] reported that MSC treatment had good efficacy. The sample size from these included RCTs was small, and the feasibility of the evidence might not be better than that from the self-controlled trials. However, larger sample RCTs should be conducted in the future.

In the past decades, other meta-analyses have confirmed that MSCs might be good agents to treat some diseases. Shi et al. [31] conducted a meta-analysis to detect the efficacious clinical therapy of MSC for the treatment of ulcerative colitis, including 8 animal and 7 human trials, and reported that MSC treatment reduced the disease activity index when compared with that in the control group in mice, and compared with the control group, the healing rate of patients treated with MSC was notably elevated. Fan et al. [32] performed a meta-analysis including nine investigations for MSC in the treatment of heart failure and reported that the overall rate of death was reduced in the MSC treatment group, which suggested that therapy of MSC was effective for heart failure by improving the exercise and prognosis capacity. Yubo et al. [33] conducted a study using a meta-analytical method to evaluate the therapeutic efficacy and safety of MSC therapy for patients with knee osteoarthritis and included eleven eligible studies including 582 patients with knee osteoarthritis, and the results showed that the MSC therapy could notably reduce the visual analogue scale score and increase the International Knee Documentation Committee scores compared with those of controls after a 24-month follow-up. The researchers concluded that MSC transplantation therapy was safe and had great potential to become an efficacious clinical therapy for patients with knee osteoarthritis. Our meta-analysis also reported that MSCs might be a promising therapeutic agent for patients with SLE.

However, there were some limitations in our study. The sample size of the included studies was small, and longer-term endpoints were needed. The severity of the patients' disease was inconsistent, and the basic regimen for the SLE patients was different. Furthermore, the dose of MSC administered varied from the number of repeats to the absolute dose amount to a per-kg dosage. These factors may have caused our results to be unstable.

## 5. Conclusions

The RCT results indicated that the MSC group had lower proteinuria at 3 months and 6 months, and the MSC group displayed lower SLEDAI at 2 months and 6 months. Furthermore, the MSC group showed a lower rate of adverse events. In case series trials, the results indicated that the MSC group had lower proteinuria at 1 month, 2 months, 3 months, 4 months, 6 months, and 12 months. MSCs might be a promising therapeutic agent for patients with SLE. However, more studies with longer-term end points and larger sample sizes should be designed and conducted to identify additional and robust patient-centred outcomes in the future.

## Figures and Tables

**Table 1:** Characteristics of the studies included in this meta-analysis.

Author, year	Study type	Basic regimen	Patient characteristics	MSC	MSC dose	Infusion	Endpoint	Adverse events	Effectiveness
Yang, 2014	RCT	GC+CTX	Refractory SLE	UC-MSC	3 × 10^7^, once	IV	SLEDAI, proteinuria, Scr, serum albumin, C3, C4	—	Yes
Zeng, 2016	RCT	GC+MMF	II-IV type of LN	UC-MSC	1 × 10^6^/kg, 3-5 times	Renal artery	SLEDAI, proteinuria, Scr, serum albumin, C3, C4, BUN	—	Yes
Tang, 2016	RCT	GC+MMF+CTX	IV type of LN	UC-MSC	5 × 10^7^, twice	IV	SLEDAI, proteinuria	T (*n* = 2): 1 case with upper respiratory tract infection, 1 case of right thigh abscess; C (*n* = 1): 1 case with upper respiratory tract infection	No
Deng, 2017	RCT	GC+CTX	IV type of LN	UC-MSC	2 × 10^8^	IV	Proteinuria, Scr	T (*n* = 2): one with leucopenia and pneumonia together with subcutaneous abscess. Another with severe pneumonia; C (*n* = 2): one patient with stroke and another with ascites of unknown cause	No
Gu, 2014	Self-control	GC+CTX/MMF	Refractory SLE	BM-MSC, UC-MSC	1 × 10^6^/kg, once	IV	SLEDAI, proteinuria, Scr, BUN	Enteritis, diarrhea, transient increase of serum creatinine, herpes virus infection. But, none of them were considered to be related to MSC infusion	Yes
Zhu, 2016	Self-control	GC+CTX/MMF	Refractory SLE	UC-MSC	5 × 10^7^, twice	IV	SLEDAI, Scr, BUN, C4	Adverse event was not found	Yes
Li, 2016	Self-control	GC+CTX	III-IV type of LN or with type V	UC-MSC	1 × 10^6^/kg-2 × 10^6^/kg, 4 times	IV	SLEDAI, proteinuria, Scr, C3, C4	Two cases of fever, 2 cases of diarrhea, 1 case of vomiting, 1 case of pruritus	Yes
Qiu, 2016	Self-control	GC+CTX/MMF	Refractory SLE	UC-MSC	1 × 10^6^/kg, once	IV	SLEDAI, proteinuria, C3	Adverse event was not found	Yes
Bai, 2017	Self-control	GC+CTX/MMF	Refractory SLE	UC-MSC	1 × 10^6^/kg, 3-5 times	IV	SLEDAI, proteinuria, C3, C4	One patient with headache, nausea, and vomiting during each stem cell infusion	Yes
Wen, 2019	Self-control	GC+CTX/MMF/LEF/HCQ	Refractory SLE	BM-MSC, UC-MSC	1 × 10^6^/kg, once	IV	SLEDAI	—	Yes

Note: RCT: randomized controlled trail; BM-MSC: bone marrow-derived mesenchymal stem cells; UC-MSC: umbilical cord-derived mesenchymal stem cells; TAC: tacrolimus; GC: glucocorticoids; CTX: cyclophosphamide; IV: intravenous; MMF: mycophenolate mofetil; HCQ: hydroxychloroquine; LEF: leflunomide; SLE-DAI: systemic lupus erythematosus disease activity index; LN: lupus nephritis; T: MSC group; C: control group.

**Table 2:** Meta-analysis of the efficacy of MSC in the therapy of patients with lupus nephritis (RCT).

Indicators	Time point	Studies	*Q* test	Model	OR/WMD	*P*
		Number	*P* value	Selected	(95% CI)	
Proteinuria	2 months	1	—	Fixed	-1.74 (-5.00, 1.52)	0.30
	3 months	1	—	Fixed	-0.92 (-1.05, -0.79)	<0.00001
	6 months	2	0.84	Fixed	-2.00 (-3.81, -0.19)	0.03
	12 months	2	<0.00001	Random	-0.46 (-1.37, 0.45)	0.33

Scr	3 months	1	—	Fixed	-2.52 (-8.53, 3.49)	0.41
	6 months	1	—	Fixed	3.92 (-8.55, 16.39)	0.54
	12 months	2	0.05	Random	-0.74 (-14.04, 12.56)	0.91

Serum albumin	3 months	1	—	Fixed	7.85 (5.93, 9.77)	<0.00001
	12 months	2	0.15	Fixed	0.94 (-0.53, 2.40)	0.21

C3	3 months	1	—	Fixed	0.28 (0.16, 0.40)	<0.00001
	12 months	2	0.02	Random	0.36 (-0.08, 0.79)	0.11

C4	3 months	1	—	Fixed	-0.01 (-0.04, 0.02)	0.46
	12 months	2	0.31	Fixed	-0.01 (-0.03, 0.01)	0.39

SLEDAI	2 months	1	—	Fixed	-6.25 (-9.04, -3.46)	<0.0001
	3 months	1	—	Fixed	-0.89 (-2.19, 0.41)	0.18
	6 months	1	—	Fixed	-4.25 (-6.78, -1.72)	0.001
	12 months	2	0.004	Random	-1.00 (-3.13, 1.14)	0.36

Adverse events	—	2	0.66	Fixed	0.26 (0.07, 0.89)	0.03

**Table 3:** Meta-analysis of the efficacy of MSC in the therapy of patients with lupus nephritis (self-control).

Indicators	Time point	Studies	*Q* test	Model	WMD	*P*
		Number	*P* value	Selected	(95% CI)	
Proteinuria	1 month	2	0.83	Fixed	-0.69 (-1.02, -0.36)	<0.0001
	2 months	2	0.46	Fixed	-1.51 (-2.40, -0.63)	0.0008
	3 months	3	<0.00001	Random	-1.25(-2.00, -0.51)	0.001
	4 months	1	—	Fixed	-2.04 (-3.00, -1.08)	<0.0001
	6 months	5	0.06	Random	-1.56 (-2.14, -0.98)	<0.00001
	12 months	2	<0.00001	Random	-1.82 (-2.96, -0.67)	0.002

Scr	1 month	3	0.32	Fixed	-7.28 (-21.97, 7.41)	0.33
	2 months	2	0.0006	Random	-59.18 (-166.92, 48.56)	0.28
	3 months	3	<0.00001	Random	-75.13 (-187.01, 36.76)	0.19
	4 months	1	—	Fixed	-10.25 (-25.34, 4.84)	0.18
	6 months	2	0.72	Fixed	-14.08 (-28.09, -0.07)	0.05
	12 months	2	0.88	Fixed	-30.00 (-38.89, -21.10)	<0.00001

BUN	1 month	1	—	Fixed	-610.6 (-835.84, -385.36)	<0.00001
	2 months	1	—	Fixed	-758.4 (-960.42, -556.38)	<0.00001
	3 months	3	<0.00001	Random	-21.31 (-46.58, 3.97)	0.10
	12 months	2	0.05	Random	-4.14 (-7.89, -0.39)	0.03

C3	1 month	2	0.69	Fixed	0.15 (0.06, 0.24)	0.0006
	2 months	2	0.70	Fixed	0.25 (0.17, 0.33)	<0.00001
	3 months	3	<0.00001	Random	0.37 (-0.01, 0.76)	0.06
	4 months	1	—	Fixed	0.33 (0.13, 0.53)	0.001
	6 months	3	0.009	Random	0.23 (0.06, 0.39)	0.006
	12 months	1	—	Fixed	0.96 (0.88, 1.04)	<0.00001

C4	1 month	2	0.51	Fixed	0.02 (-0.01, 0.04)	0.25
	2 months	2	1.00	Fixed	0.05 (0.02, 0.08)	0.0001
	3 months	2	0.04	Random	0.11 (0.07, 0.15)	<0.00001
	4 months	1	—	Fixed	0.07 (0.04, 0.10)	<0.0001
	6 months	2	0.009	Random	0.06 (-0.02, 0.14)	0.15
	12 months	1	—	Fixed	0.24 (0.22, 0.26)	<0.00001

SLEDAI	1 month	5	0.02	Random	-3.83 (-5.42, -2.23)	<0.00001
	2 months	2	0.15	Fixed	-4.38 (-6.24, -2.51)	<0.00001
	3 months	4	0.88	Fixed	-5.45 (-6.19, -4.72)	<0.00001
	4 months	1	—	Fixed	-6.35 (-8.27, -4.43)	<0.00001
	6 months	6	0.87	Fixed	-7.20 (-7.99, -6.42)	<0.00001
	12 months	3	0.51	Fixed	-8.06 (-8.79, -7.33)	<0.00001

## Data Availability

The data supporting this meta-analysis are from previously reported studies and datasets, which have been cited. The processed data are available from the corresponding author upon request.
